# Real-Time Cardiac Beat Detection and Heart Rate Monitoring from Combined Seismocardiography and Gyrocardiography

**DOI:** 10.3390/s19163472

**Published:** 2019-08-08

**Authors:** Yannick D’Mello, James Skoric, Shicheng Xu, Philip J. R. Roche, Michel Lortie, Stephane Gagnon, David V. Plant

**Affiliations:** 1 Department of Electrical and Computer Engineering, McGill University, Montreal, QC H3A 2T5, Canada; 2 MacDonald, Dettwiler and Associates Corporation, Ottawa, ON K2K 1Y5, Canada

**Keywords:** seismocardiography, gyrocardiography, electrocardiography, vibrational cardiography, heart rate monitoring, electromechanical cardiac activity, wearable sensors

## Abstract

Cardiography is an indispensable element of health care. However, the accessibility of at-home cardiac monitoring is limited by device complexity, accuracy, and cost. We have developed a real-time algorithm for heart rate monitoring and beat detection implemented in a custom-built, affordable system. These measurements were processed from seismocardiography (SCG) and gyrocardiography (GCG) signals recorded at the sternum, with concurrent electrocardiography (ECG) used as a reference. Our system demonstrated the feasibility of non-invasive electro-mechanical cardiac monitoring on supine, stationary subjects at a cost of $100, and with the SCG–GCG and ECG algorithms decoupled as standalone measurements. Testing was performed on 25 subjects in the supine position when relaxed, and when recovering from physical exercise, to record 23,984 cardiac cycles at heart rates in the range of 36–140 bpm. The correlation between the two measurements had *r*^2^ coefficients of 0.9783 and 0.9982 for normal (averaged) and instantaneous (beat identification) heart rates, respectively. At a sampling frequency of 250 Hz, the average computational time required was 0.088 s per measurement cycle, indicating the maximum refresh rate. A combined SCG and GCG measurement was found to improve accuracy due to fundamentally different noise rejection criteria in the mutually orthogonal signals. The speed, accuracy, and simplicity of our system validated its potential as a real-time, non-invasive, and affordable solution for outpatient cardiac monitoring in situations with negligible motion artifact.

## 1. Introduction

Cardiovascular disease (CVD) is the largest contributor to global mortality rates [1]. The global prevalence of CVD-related incidents has motivated the medical community to seek solutions that reduce the onus of regular check-ups on health care systems. This approach of ‘prevention through prediction’ [2] highlights a need for accessible cardiac monitoring solutions [3], especially since preventive care has the potential to reduce global mortality rates by millions and economic losses by trillions [4]. Certain symptoms of cardiac failure or arrest are identifiable prior to its occurrence [5]. Using recognized patterns and anomalous behavior in cardiac activity [6,7], intuitive sensing and signal processing algorithms could be used to predict the incidence of a stroke or heart attack [8,9] by identifying the early signs of potentially fatal problems before their onset [10,11]. Real-time, continuous health monitoring could improve the accuracy of medical treatments and hasten diagnoses through the development of algorithms that connect physiological signals to health state trajectories. A growing availability of evidence-based analysis from biometric databases improves the potential of such monitoring algorithms to classify meaningful trends [12]. A key component of our work involves the development and assessment of a real-time algorithm that interprets certain cardiac function by recognizing patterns in cardiography signals.

Cardiac monitoring is an indispensable aspect of health evaluation. Heart rate is recognized as a representative measurement of cardiac function [13]. Despite a wide variety of available techniques for heart rate measurement, the task is nontrivial outside of medical scenarios such as in the field or at home. Photoplethysmography, while easy to use, is difficult to extract intra-beat information from a distal site and is sensitive to motion artifact [14]. Electrocardiography (ECG) can be difficult to implement by an untrained user in a domestic environment, and the electrodes cause irritation over prolonged use [15]. This automatically rules out impedance cardiography [16], which requires a higher level of system complexity than ECG. Additionally, ECG provides a measurement of the surface electric activity pertaining to the cardiovascular system, from which cardiac mechanics are assumed but not obtained [17]. Using mechanical cardiography, cardiac-induced mechanical vibrations have been measured through their influence on the body. Ballistocardiography provides a measure of the impact of ventricular ejection on the displacement of the entire body [18], which is inherently not portable. Alternatively, phonocardiography offers a reliable, portable method to detect the acoustic waves produced by heartbeats [19] albeit with limited information due to the fact that primary heart sounds are an indirect consequence of valve operation. Cardiac mechanics generate predominantly compression waves that diffuse through the chest [20]. These cardiac-induced vibrations produce accelerations of the sternum that can be detected as a seismocardiography (SCG) [18] signal. Initially demonstrated in 1961 [21], the method did not gain much traction due to the cumbersome size of accelerometer equipment. Its resurgence was motivated by advances in micro-electro-mechanical system (MEMS) based motion tracking technology that integrated the accelerometer and gyroscope into a miniaturized inertial measurement unit (IMU) [18]. This recent availability of an integrated, coupled gyration signal motivated research in gyrocardiography (GCG) [22] as a complementary measurement. Vectorially, the combined motion from all six mutually orthogonal degrees of freedom represented a basis set for vibrational analysis at the sternum. This bifocal view of coupled SCG and GCG hence delivered a comprehensive vibrational cardiography (VCG) measurement using a single sensor.

Cardiac activity originating in action potentials (recorded by ECG) induces mechanical compressions that generate low frequency vibrational waves. These vibrational waves diffuse through the chest and can be detected at the sternum. Three-dimensional (3D) linear acceleration is detected as seismocardiography (SCG) [21,23] and the coupled, 3D angular gyration as gyrocardiography [24]. While there is yet no direct model describing the causal relationship between sternal vibrations and the deformation of cardiac walls [24,25], key events in the cardiac cycle show a clear correlation with fiducial features in both SCG and GCG waveforms [22]. Their fidelity has also been validated through the coherence of ensembled averages of the quasi-periodic waveforms. Considering the position of the heart in the thorax, VCG signals are strongest near the xiphoid process of the sternum [26]. At this position, accelerations caused by cardiac vibrations are projected outward along the dorsoventral axis, and consequently rotate the sensor in the two spatially coupled gyration axes. Up to 60% of cardiac vibrational energy is contained in the gyration signal [27], which suggests a higher noise rejection ratio than acceleration data. As the two measurement methods are mutually orthogonal, they are inherently susceptible to different noise characteristics, which enables a deeper analysis when combining the information from both. GCG offers the potential for novel insights into cardiac fiducial points [22,28], higher fidelity for certain types of motion artifact [29,30,31], and can assist in SCG beat detection using kinetic energy envelopes [30,32]. In this paper, we have modified our previously developed autocorrelated differential algorithm (ADA) [33] to process GCG in parallel with SCG and thereby exploit the content-rich information in VCG signals. The system was also expanded to reference with ECG, thereby demonstrating an affordable tool for electro-mechanical cardiography.

The accessibility of VCG makes it an attractive solution for cardiac monitoring especially in non-clinical and everyday scenarios. Recent work has shown the use of smartphones for cardiac analysis [34,35,36], despite a strong susceptibility of signal degradation from motion artifacts. Biomechanical movement, external impacts, and involuntary spasms are rife in VCG sensor data [26]. The strength and similarity of their noise profiles to the signal itself emphasizes a need for robust, accurate signal processing algorithms for real-time waveform analysis [37]. However, current algorithms are complex [38,39,40], or require a large amount of initial reference data [41], which renders them infeasible for everyday use. Algorithms that rely on training data sets [42,43] are limited in versatility, customizability, and efficiency. A common workaround for motion artifact cancellation in VCG is by correlation with ECG [44,45,46,47,48], that is, by leveraging the reliability of R-peak detection to inform mechanical aortic opening (AO) detection. However, the integration of ECG doubles the overall system complexity requiring unique hardware, algorithms, and clock synchronization. We have therefore developed a VCG-based measurement algorithm that can be referenced with ECG if necessary. Our analysis exploited the quasi-periodicity of consecutive cardiac cycles in VCG waveform morphology. Cardiac cycles were identified by correlating with previous cycles in a short-term learning-based approach. However, a known limitation of autocorrelation-based algorithms is effective signal averaging that limits heart rate variability (HRV) detection. We addressed this issue by windowing the incoming signal, measuring the beat-to-beat intervals within a window, and consequently HRV. The robustness of the approach was evaluated on declining heart rates during post-exercise recovery, which represented a challenging test scenario due to the existence of higher, exerted respiration metrics coupled with the lack of a baseline heart rate (HR). Our work contributes to a global effort toward real-time, non-invasive, and inexpensive cardiac monitoring solutions. By using standard MEMS and microelectronic systems, we present a low-cost setup that delivered VCG analysis as well as referencing with ECG.

The structure of the paper is as follows. The methods are described in Section 2, which includes the system configuration for data acquisition, and the protocols used to perform biometric measurements on human subjects, followed by the algorithm workflow, and the metrics used to characterize performance. The architecture of the ADA that was extended for VCG analysis is described in Section 3, which consists of pre-processing, heart rate measurements on SCG and GCG signals, their application to beat identification, and the of feedback loops and redundancy incorporated into decision-making. The experimental results in Section 4 consist of a demonstration of heart rate monitoring at rest and during post-exercise recovery as well as a comparison of the accuracy of the ADA when applied on SCG, GCG, and VCG in reference to an ECG benchmark. We have used this heart rate measurement in the identification of AO peaks and evaluated the accuracy of this detection scheme. Finally, we assess the potential of the system for real-time monitoring by characterizing its accuracy and computational time (i.e., maximum refresh rate) for different sampling frequencies. We discuss the significance, advantages, constraints, and limitations of our results in Section 5. The paper is concluded in Section 6 with a summary of the main results.

## 2. Methods

### 2.1. System Configuration

**Figure 1:**
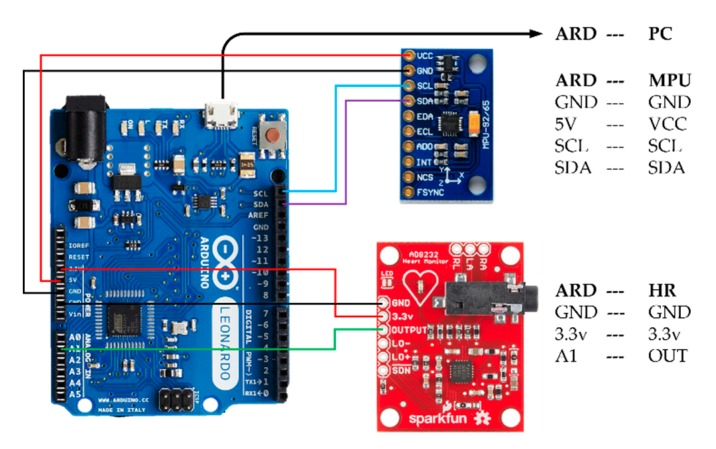
System configuration between the MPU-9250 [49], AD8232 [50], and Arduino Leonardo [51].

The system was assembled from commercial, off-the-shelf components. Cardiac-induced vibrations were detected by an inertial measurement unit (IMU) placed at the xiphoid process of the sternum. The IMU sensor chosen was a nine-axis InvenSense Motion Processing Unit™ (MPU) 9250 (San Jose, CA, USA) consisting of a MEMS gyroscope and accelerometer, along with a digital compass that was not used in this work. The sensitivity of the MPU-9250 accelerometer and gyroscope were set by fixing the operate range at ±2 g and ±250 °/s respectively. ECG data was recorded simultaneously by a SparkFun AD8232 Single Lead Heart Rate Monitor (Niwot, CO, USA). Both sensors were integrated using an Arduino Leonardo microcontroller. As shown in the system configuration diagram of Figure 1, the microcontroller was strapped to the torso and transmitted sensor signals serially to a computer via USB cable, which was also used to power the system. The sampling rate of the Arduino was approximately 250 Hz. Data acquisition and signal processing were controlled by a custom built, Matlab-based graphic user interface (GUI). As an external reference for the system, three heart rate measurements were recorded using an Omron 10 Series oscillometric blood pressure monitor at the brachial artery over a period of approximately three minutes during testing. The measurements obtained by the cuff were entered into the GUI manually during data acquisition.

### 2.2. Measurement Protocol

**Figure 2:**
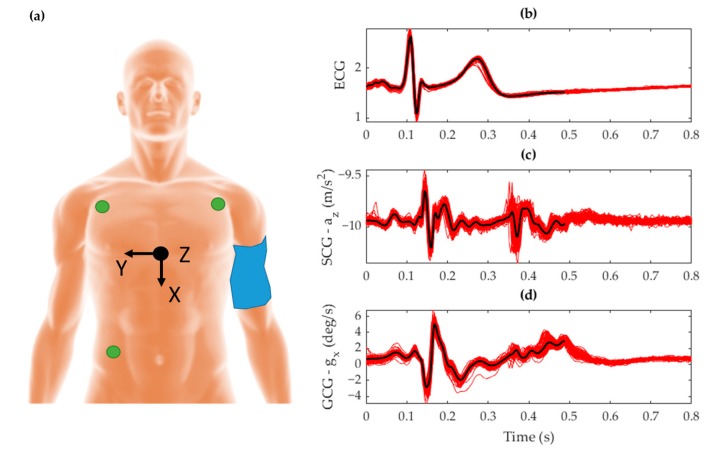
(**a**) Placement of the inertial measurement unit (IMU) sensor (black) with its orientation represented by the Cartesian reference axis, the electrocardiography (ECG) electrodes (green) attached at the extremities of the torso, and the cuff sphygmomanometer (blue) strapped around the upper left arm with the subject lying supine. The corresponding signal morphology is shown for (**b**) ECG voltage, (**c**) seismocardiography (SCG) Z-axis acceleration, and (**d**) gyrocardiography (GCG) X-axis gyration. The plots show ensembled averages (black) of the raw signals (red) that were temporally aligned to the time signature of the R-peak over a window of 100 beats.

Regarding sensor placement, the MPU-9250 was attached to the skin at the xiphoid process of the sternum [52]. The positive X, Y, and Z-axes of the accelerometer were oriented downward along the longitudinal axis, right along the frontal axis, and outward along the dorsoventral axis of the body, respectively. Gyroscope co-ordinates followed the right-hand rule for rotation about these axes. Electrodes measuring cardiac electrical activity for ECG were placed in an Einthoven triangle [53] on the torso. This placement is shown in Figure 2a for the VCG IMU, ECG electrodes, and sphygmomanometer cuff. The corresponding signal morphology obtained from this placement is shown in Figure 2b–d for ECG, SCG, and GCG respectively.

Testing was conducted with approved protocols in accordance with the Review Ethics Board at McGill University. The biometric signals of 25 male subjects between the ages of 20 and 30 years old that had no known cardio-respiratory ailments were measured. The protocol consisted of two tests, of approximately seven minutes in duration. The first test involved each subject resting supine. One minute after the start of ECG and VCG data acquisition, sphygmomanometer cuff monitor was activated. Three consecutive pulse rate measurements were performed using the cuff during the 7 min duration of the testing cycle. This measurement provided baseline heart rate of the participants while they were at rest. Following this test, the subjects performed a high intensity floor exercise known as the mountain climber. The exercise was performed without any warm up activity so that subjects underwent sufficient cardiovascular exertion to elevate their heart rate. While the extent of exercise required for exertion is heavily dependent on interpersonal variations in fitness [54], approximately one minute of this exercise was found to induce a sufficient elevation in heart rate. The second test was conducted immediately after the subjects chose to end the exercise. Data was collected with the subject lying in the supine position following the same protocol as the previous tests.

### 2.3. Algorithm Workflow

**Figure 3:**
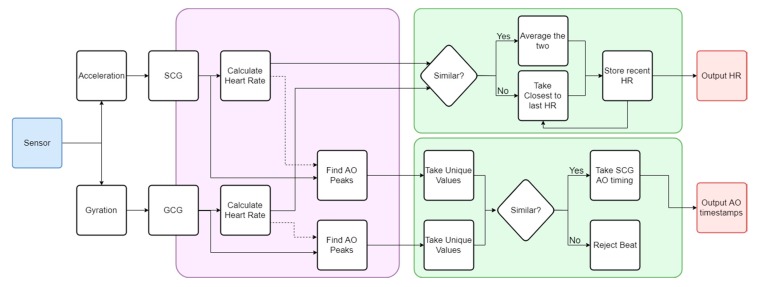
Flowchart of the autocorrelated differential algorithm (ADA) algorithm extended to include GCG, thereby enabling complete vibrational cardiography (VCG) measurements. The violet region incorporates the core ADA algorithm computed for both SCG and GCG. The green region contains the decision protocol. Key output parameters are in red.

The autocorrelated differential algorithm (ADA) developed in [33] was built to process a three-axis acceleration signal for cardio-respiratory measurement. In this work, the algorithm was extended to incorporate three additional degrees of freedom (DOF) corresponding to the angular rotation of the sensor. The final algorithm architecture consisted of a multi-input/output configuration with parallel processing for acceleration and gyration signals, and its workflow shown in Figure 3. For the purpose of this study, respiratory measurements were filtered from final verification.

Fundamentally different morphologies in the SCG and GCG signals seen in Figure 2 imposed a requirement on the algorithm that the waveforms be analyzed independently. In this context, both signals were processed separately in the ADA as can be seen in the violet region of Figure 3, with the algorithm described in Section 3.2. Two similar streams of cardiac-related biometric information were generated. Any divergences between these two results were attributed to noise and motion artifacts detected during testing, as well as the effectiveness of the waveform analysis. They were addressed by the decision-making algorithms shown in the green box of Figure 3 that compared both output heart rates using the specific rejection criteria as given in Section 3.3. The final result was recorded as a heart rate measurement with corresponding beat identification, as represented by the red boxes of Figure 3. Within each signal, individual beats were identified by using the heart rate measurement as an indicator of the beat-to-beat interval. This stage of the ADA was developed to extract the timestamp corresponding to each heartbeat as detailed in Section 3.3. A beat was classified as the timestamp of the aortic opening (AO) fiducial feature in the SCG waveform, which is a commonly used surrogate for the ECG R-peak [55]. The independently calculated SCG and GCG beat detection streams were merged in a separate decision-making process described in Section 3.3.

### 2.4. Performance Characterization

The performance of the algorithm was evaluated against ECG, which is a recognized standard for cardiac activity detection [3,56]. As a sanity check, the acquisition from the system was also verified with an external sphygmomanometer. The Pan-Tompkins algorithm was used to detect ECG R-peaks [57]. These peaks indicated the beat-to-beat (BTB) interval represented by the R–R interval, from which heart rate was extracted and processed similar to the ADA results. The algorithm was benchmarked in two stages. Initially, the heart rate produced by the ADA was compared with the reference ECG result after averaging the results over a 10 s window as detailed in Section 3.1. A squared Pearson’s correlation coefficient (*r*^2^) was used to determine the linear correlation between both results. The second stage of the characterization involved evaluation of the beat detection methods. As this measurement was quantized to each beat, a more rigorous statistical approach was appropriate.

The resulting AO timestamps of the ADA were compared with R-peak timings obtained using the Pan-Tompkins algorithm. A true positive (TP) beat was indicated by AO peak detection within 250 ms from an R-peak timestamp, which was optimized experimentally. Any AO timestamps located outside the range of an R-peak timestamp were labeled as false positives (FP). Locations where an R-peak was identified without a corresponding AO timestamp within range were labeled as false negatives (FN). Due to the implementation of this algorithm for beat identification, true negative (TN) points were unnecessary. Using these metrics, the following statistical tools were used to evaluate beat identification accuracy. The sensitivity of detection was determined by the true positive rate (TPR), which calculated the proportion of correctly identified beats as,

(1) $$\mathrm{TPR}=\frac{\mathrm{TP}}{\mathrm{TP}+\mathrm{FN}}.$$

Similarly, the precision was measured using the positive prediction rate (PPV), which determined the likelihood of a detected beat to be correct,

(2) $$\mathrm{PPV}=\frac{\mathrm{TP}}{\mathrm{TP}+\mathrm{FP}}.$$

Reflecting the algorithm design priority to measure heart rate, the identified beats were further converted into an instantaneous heart rate measurement from the inverse BTB. BTB was calculated from the AO–AO intervals of the TP beats. Hence, as a final evaluation of the beat identification results, the agreement between this quantized HR and its ECG reference (derived from the RR-interval) was evaluated using the same squared Pearson’s correlation coefficient (*r*^2^) mentioned previously.

## 3. Algorithm Architecture

The previously developed ADA algorithm [33] was extended to analyze GCG signals and thereby the entire VCG parameter space. Its accuracy was further validated by applying the measured HR to inform AO peak identification. In this way, instantaneous as well as averaged heart rate could be calculated. The following subsections describe the architectural features and highlights of the algorithm in the context of its upgrade for the purpose of VCG beat identification.

### 3.1. Heart Rate Calculation

In order to emulate a real-time monitoring environment and accommodate a user-friendly scheme, the algorithm was set to deliver measurements with a 1 Hz refresh rate. This implied that a measurement was performed on every 1 s of incoming data. The refresh rate was intended to be application-specific albeit with an upper limit of 0.088 s representing the computational time per measurement as explained in Section 4.3. Similar to the ADA, each measurement analyzed the most recent 10 s of data from the acquired cardio-mechanical signal to calculate heart rate. Temporal inconsistencies in the data were reorganized into a uniform sampling rate of 200 Hz using pchip interpolation on each axis. Intrapersonal and heart rate variability were accounted for via a windowing approach where multiple window sizes containing the most recent n-seconds of data (where n∈[2,10]) were processed independently. The data were filtered by a high-pass brick wall filter with a cutoff of 0.4 Hz. A heart rate calculation was conducted on each segment that corresponded to a specific axis and window size.

Within each window, the waveform analysis was conducted in three phases and was designed to be agnostic to the window size or any HRV. Assuming a consistent sensor placement and orientation, the $a_{Z}\;$and $g_{X}\;$axes exhibited a quasi-periodic waveform similar to the ensembled averages in Figure 2. Here, the first phase emphasized amplitude variations corresponding to the first heart sound (S1) based on the expected waveform morphology in each axis. These variations were exploited in a custom built, irreversible transfer function that amplified the variations in a window and was named VarWin. By scanning for large oscillations in amplitude within a range of approximately 250 ms of each data point, the VarWin functionality enhanced the amplitudes and widths of any characteristic variations in each axis that indicated the occurrence of a S1 related event. Hence a peak in the VarWin output represented the occurrence of both a local maximum and a local minimum within 250 ms window. In the SCG waveform, this occurred during the S1 heart sound caused by the AO and IC fiducial points. A signal amplification around the AO points exclusively, along with smoothing over the remaining elements of the S1 sound, resulted in a higher correlation between beats. In the second phase, autocorrelation of the resulting waveform produced much stronger harmonics than would otherwise have been detected on a raw signal due to systemic noise factors such as HRV and motion artifact. Finally, the waveform was filtered to eliminate artifacts from incorrect overlaps or artificial peaks. Using thresholds within an acceptable range for human subjects, the interval between harmonics in the autocorrelation was judged from the BTB and the amplitudes of the AO peaks were filtered from the aortic closing (AC) peaks. Results from all the windows were concatenated by a weighted mean with weights that were inversely proportional to the window size, similar to the ADA. Therefore, the algorithm produced one final heart rate per time step, based on the last 10 s of SCG and GCG data with a priority given to the most recently acquired samples.

### 3.2. Beat Identification

**Figure 4:**
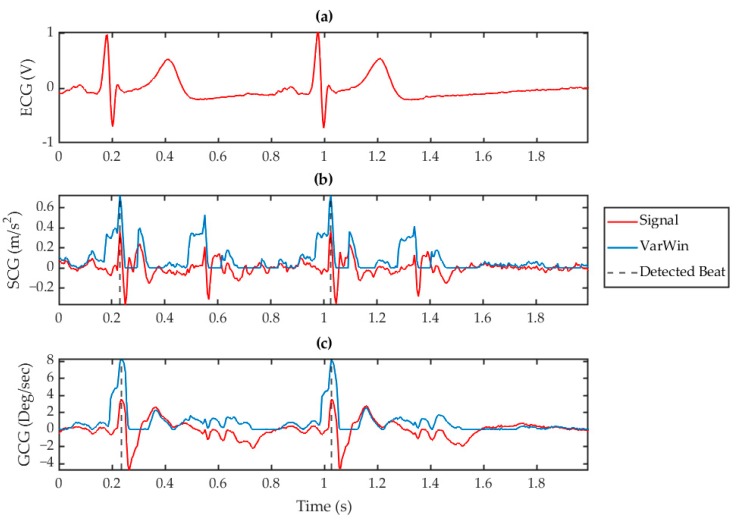
Processed signals for (**a**) ECG, (**b**) SCG (red) with its corresponding VarWin waveform (blue), and (**c**) GCG (red) with its corresponding VarWin waveform (blue) for a 2 s window. The vertical, dashed grey lines indicate the detected peaks using this technique.

Fiducial features of the SCG and GCG waveforms have been found to correlate with the timing of known cardiac events [24,25]. Most intra-beat annotation schemes rely on the identification of the AO event due to its direct relationship with the ECG R peak through the cardiovascular electro-mechanics during the S1 sound. The AO peak is commonly used as a surrogate for the ECG R peak in heart rate evaluation and S1 beat identification. It can therefore be inferred that the development of a robust AO detection algorithm is a key aspect toward the use of VCG in heart rate monitoring applications. However, traditional amplitude thresholding or feature based approaches are highly susceptible to variations in VCG morphology from orientational, systemic, or situational differences. In the ADA, feature recognition was facilitated by amplifying the morphology between intra-beat fiducial points with respect to the rest of the waveform through a modified version of the VarWin function. For SCG, the waveform was amplified in the region around the S1 and S2 sounds corresponding to the AO and MO points, respectively. The modified VarWin window only considered variations in points that occurred after the current point. Conversely, features occurring directly after a local minimum were also suppressed. The result of this technique was to amplify the differential between the AO and IC points, while suppressing conflicting RE points. This process is visualized in Figure 4b, in which the filtered SCG wave is transformed to an enhanced waveform with amplified AO peaks within a 2 s window. A similar process was used for feature recognition on the GCG waveform by using the gyrational velocity of the $-g_{X}$ axis. As can be seen in Figure 4c, the GCG fiducial point corresponding to the SCG AO point was amplified although the amplification of the S2 region in the $g_{X}$ processed waveform was comparatively less than in the $a_{Z}$ signal. These different VarWin outputs between SCG and GCG reduced the crosstalk of the S2 region and further validated the benefit of drawing on both methods simultaneously.

These clearly defined peaks facilitated a high accuracy in peak detection and identification. The minimum spacing between detected peaks was set at a threshold above the calculated heart rate for the given window, that is, at a minimum of 75% of the average BTB interval. In this manner, multiple AO peaks were detected within each window. The list of timestamps corresponding to the AO peaks were cross verified using decision-making algorithms, for which the redundancies between multiple windows was found to significantly boost overall accuracy.

### 3.3. Consolidation of SCG and GCG

A direct consequence of measuring heart rate from the demultiplexed VCG waveform was commonly occurring redundancies and inconsistencies in measurement results between the vibrational axes. Although the VCG waveforms shared the same origin, the mutually orthogonal signals gleaned from SCG versus GCG inherently produced different measurements. A decision-making algorithm was therefore developed to interpret and filter the results using comparison followed by a feedback loop.

Both auto-correlated heart rate calculations from the $a_{Z}$ and $g_{X}$ axes, and the heart rate from the previous measurement instance were provided as an input to this decision-making algorithm. A successful dual measurement was interpreted as both SCG and GCG based calculated heart rates being within a tolerance of 10 bpm of each other, thereby ensuring a cross-verified heart rate. In this case, the two results were averaged in the final HR output measurement. Alternatively, a difference of more than 10 bpm in the measured heart rates indicated a failure in one of the measurement methods. In this scenario, the previous heart rate measurement was incorporated in the comparison as a third input to interpret the current one. This exerted a reliance on the quasi-periodicity of the cardiac cycle in the sense that the HR reading was assumed to be relatively consistent between sequential measurements. As the refresh cycle was set to be 1 s, the previous measurement was considered a valid verification of the obtained results because it reflected the last known heart rate. Both results were compared to this reference and the closer result was interpreted as the final measurement.

Averaged HR was then used to calculate the instantaneous HR through individual beat identification. As described in Section 3.2, the AO point in the $a_{Z}$ axis was used as a timing indicator of the S1 heartbeat for each window. Here, the same AO timestamp was detected across multiple windows and these the timestamps were consolidated into a single array. A decision-making algorithm was used to filter duplicate and invalid points. We note that while the first peak in the SCG waveform has been correlated with the aortic opening of the heart [25], an equivalent feature in the $g_{X}$ waveform still lacks clinical correlation with the actual AO point [24]. However, within a small range of variability, this peak has been found to occur consistently within the first heart sound [24]. The $g_{X}$ waveform was therefore used to aid the classification of AO points in the $a_{Z}$ waveform. Using both independently detected sets of peaks from each waveform, the timings of all unique points were compared between both signals. Only those SCG AO timestamps that existed within a tolerance of 25 ms of the timestamp of a peak in the waveform were considered valid, else they were discarded. The retention assumed that they fit the BTB interval indicated by the HR calculation in that timeframe. Hence only simultaneous peaks in both the $a_{Z}$ and $g_{X}$ waveforms were retained with the rest discarded. The final timestamps were used to provide measurements of the BTB interval, and consequently the inverse BTB as an instantaneous heart rate.

## 4. Results

### 4.1. Heart Rate Measurement

The system was tested on 25 subjects with two recordings per subject. As described in Section 2, the first recording was conducted on a resting supine subject, which provided a baseline measurement of the resting heart rate and HRV. The second recording was taken immediately after a short burst of intense cardiovascular exercise, which captured elevated cardiac activity. It allowed for a larger range of heart rates and HRV to evaluate the system limitations. A rapidly decaying heart rate further verified the feasibility of the feedback loops described in Section 3.3, and their susceptibility to bias.

**Figure 5:**
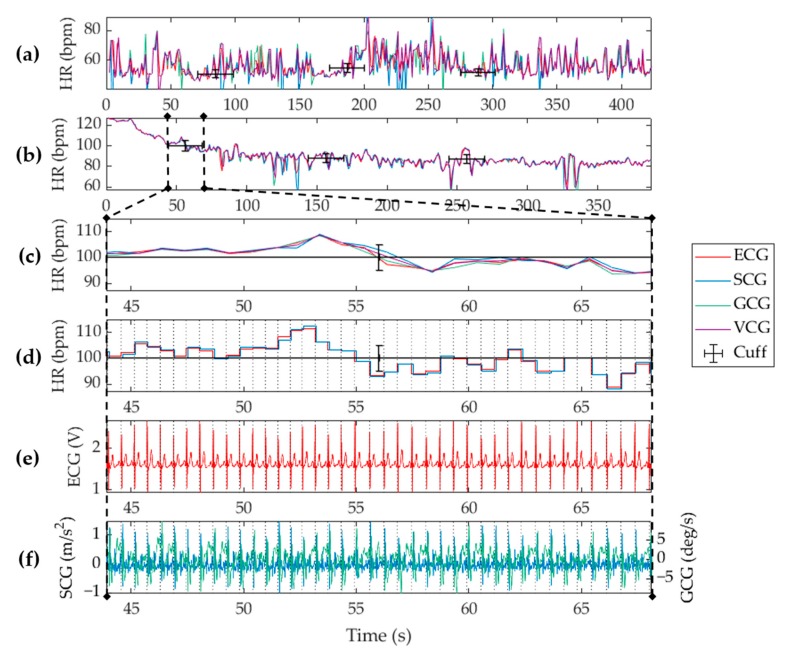
Biometric measurements from a single subject using the proposed cardiac electro-mechanical monitoring system when the subject was lying supine (**a**) at rest, and (**b**) recovering from physical exertion. Heart rate measurements obtained from ECG (red), SCG (blue), GCG (green), and VCG (purple), are shown with the cuff measurements (black) overlaid. A zoomed snippet of the dataset is shown for (**c**) the ADA-based heart rate, and (**d**) beat-to-beat (BTB)-based heart rate. Shown also are (**e**) the raw vibrational signals, and (**f**) the ECG signal. Vertical grey dotted lines show detected beats.

A total of 50 datasets were examined in this study. Four out of the fifty data sets were discarded due to poor signal quality in either the vibrational or electrical waveforms. The failures were attributed to acquisition errors from sensor displacement or interruptions in the physical connections. Analysis on each dataset was performed on a per second basis according to the stipulated refresh rate, and measurements were extracted using the algorithm described in Section 2 and Section 3. The heart rate measured for a single subject is shown in Figure 5. A full seven-minute recording of a supine subject at rest is shown in Figure 5a with the cardiac response of the same subject when recovering from exertion shown in Figure 5b. A zoomed-in timeframe of the waveforms during the first cuff deflation is shown in the remaining subplots, which display an in-depth view of the measurements that occurred within the temporal uncertainty of the Omron S10 cuff measurement. As expected, the heart rate measurement shown in Figure 5c was subject to a higher level of variability than the single value outputted by the cuff, which is why the cuff was only used as a sanity check to verify the system hardware. As a measurement reference, the ECG based heart rate measurements were correlated with those obtained from VCG analysis. We note that the windowed autocorrelation approach effectively smoothed heart rate measurements as is evident when compared to the instantaneous heart rate shown in Figure 5d. The instantaneous HR calculation showed an effective quantization to each cardiac cycle. It maximized the accuracy of the algorithm and enabled HRV tracking. However, note that this quantized HR measurement is not often implemented in scenarios such as activity tracking or fitness monitoring because such levels of cardiac activity are better indicated by an average, that is, beats per minute, due to the possibility of a high variability at any given beat. The corresponding ECG, SCG $a_{Z}$, and GCG $g_{X}$ input waveforms are also shown in Figure 5e,f for comprehension. Collectively, this figure is a snapshot of the available features of the system and its corresponding algorithms.

### 4.2. Statistical Analysis

**Figure 6:**
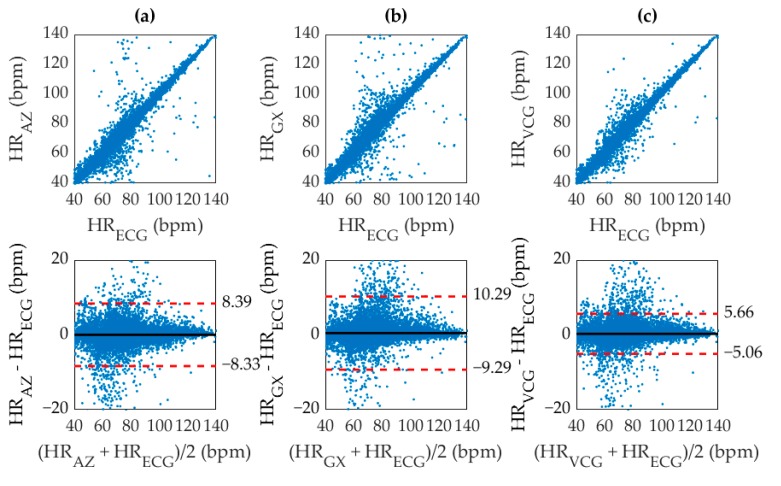
Correlation (top) and Bland Altman (bottom) plots for (**a**) SCG with *r*^2^ of 0.9485, (**b**) GCG with *r*^2^ of 0.9305, and (**c**) VCG with *r*^2^ of 0.9783. The (black, solid) mean difference, and (red, dashed) 95% limits of agreement are shown overlaid.

Inherent variations in physiological recording due to body structure, HRV, fitness, sensor placement, and other factors induce significant differences between the signals measured from different techniques. The SCG, GCG, and VCG based measurements were therefore compared with their corresponding ECG reference measurements by computing the squared Pearson’s correlation coefficient between the methods. The correlation plots can be seen in Figure 6 along with the corresponding Bland-Altman plots showing the 95% limits of agreement between results.

The *r*^2^ values for SCG, GCG, and VCG were 0.9482, 0.9305, and 0.9783 respectively. As expected, the accuracy of the ADA for SCG was higher than GCG, which was possibly due to the maturity of the algorithm for SCG analysis or simply a higher fidelity in the SCG waveform. Furthermore, the combined approach using VCG was more accurate than either SCG or GCG. The significant narrowing of the 95% limits of agreement for VCG also indicates a reduction in dispersion, which implied a lower deviation of the inaccurate values from the reference. As apparent from the distribution of Figure 6, the ADA produced very few large outliers. A majority of the discrepancies in heart rate had a deviation of less than ±5 bpm from their corresponding ECG reference. However, a large spread of points can be observed within this range due to smoothing inconsistencies between the ECG and VCG results as a result of the windowed approach. This inaccuracy was addressed by quantizing the heart rate measurement through direct AO beat detection.

**Figure 7:**
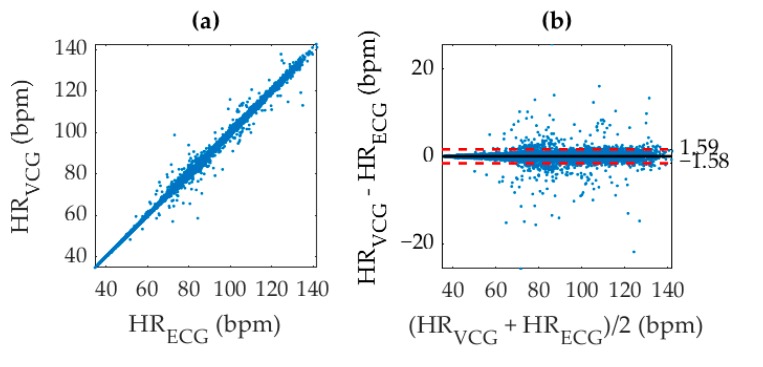
(**a**) Correlation of VCG aortic opening (AO) identification with ECG R peak identification to produce an *r*^2^ value of 0.9982, and (**b**) corresponding Bland-Altman plot. The (black, solid) mean difference, and (red, dashed) 95% limits of agreement are shown overlaid.

An identification of individual beats in the VCG waveform significantly improved accuracy since the results were no longer susceptible to sampling inconsistencies from smoothing or windowing. Evidence of this claim is in the correlation coefficient of 0.9982 for direct AO peak detection as shown in Figure 7, which was referenced with ECG R–R intervals. We acknowledge that an *r*^2^ value of 1 is physically impossible with this analysis technique due to the different electro-mechanical origin of each signal. The increased accuracy in VCG–AO detection as compared to VCG–HR measurement is an intuitive consequence of the fact that the HR measurement was used to detect AO peaks. In this analysis, the inverse BTB interval at the timestamps of each AO point was correlated with its corresponding value for the ECG R-peaks and shown in units of bpm for easy comparison with Figure 6. Hence, the averaged HR in Figure 5c essentially provided a coarse estimate that was fine-tuned to calculate the instantaneous HR measurement in Figure 5d. 

The performance of the beat detection technique was characterized using the techniques detailed in Section 2.4. The statistical parameters and their values are shown in Table 1, which quantified the accuracy of the developed system for each recording separately. An analysis of all 46 sets covered a total of 23,984 heartbeats consisting of 23,162 TP, 75 FP, and 819 FN beats. This resulted in a TPR of 96.6%, and a PPV of 99.6%. The heart rate was calculated from each of the TP beats as described in Section 3.3.

### 4.3. Computational Efficiency

**Figure 8:**
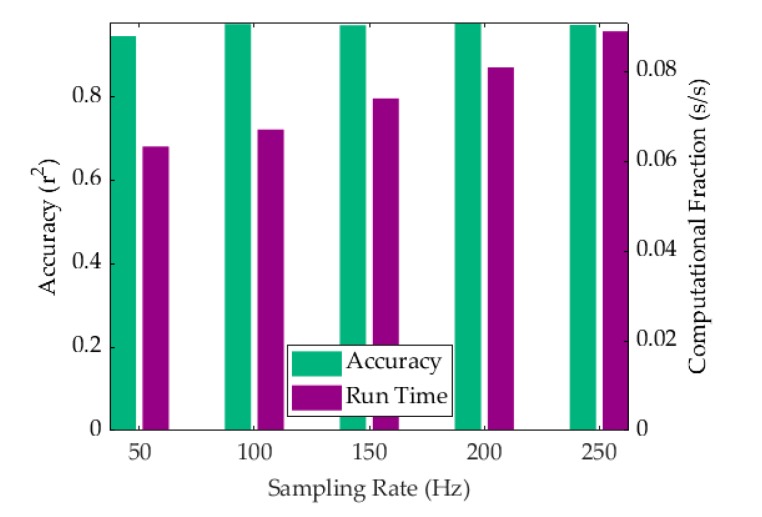
Algorithm efficiency shown as a combination of computational time per second of real-time data analyzed (purple) and the corresponding *r*^2^ accuracy (green).

The technical goal of the ADA and system development was to measure the heart rate in real-time for use as a heart-monitoring device. While the system hardware was found to be more than sufficient for the purpose of this experimental investigation, the speed of the algorithm was admittedly dependent on the computer being used to run the calculations. For slower computers, the sampling rate could be decreased to improve computational time albeit at the cost of a lower accuracy. This trade-off in efficiency was characterized in the context of the dependence of computational time and accuracy on the sampling rate. The computational time for producing the entire dataset was calculated using a Matlab timer and divided by the number of separate refreshed measurement steps to obtain the measurement time per second of real-time incoming data. The accuracy was measured using the squared Pearson’s correlation coefficient. The trade-off can be seen in Figure 8 for sampling frequencies of 50 Hz, 100 Hz, 150 Hz, 200 Hz, and 250 Hz. Interestingly, the compromise in accuracy for VCG resulted in a *r*^2^ value of 0.9456 at 50 Hz, which marked a significant improvement over SCG alone [33]. A maximum computational fraction of 0.088 s per second of data was observed at a sampling frequency of 250 Hz when using a standard desktop computer with no external programs activated. The computer used an Intel Core i7-4770K, 3.5 GHz processor with 32 GB RAM. At a sampling rate of 100 Hz, we estimated the processor requirements to maintain a refresh rate of 1 s to be well within the computational power of a commercially available Raspberry Pi unit.

## 5. Discussion

Changes in the polarization of the cardiac muscle were detected by ECG electrodes in the form of their manifestation as changes in voltage on the surface of the skin. Within the ECG signal waveform, the QRS complex represented the depolarization of the ventricles that compressed to eject blood through the aortic valve. The hydraulic opening of the valve generated compressional waves that diffused through the torso. These waves were detected by a MEMS-based IMU sensor placed at the xiphoid process of the sternum. The 3D acceleration and gyration of the sensor was analyzed by the ADA on a computer. This waveform analysis produced heart rate measurements using the ECG R-peak and the VCG AO-peak as markers of individual cardiac cycles.

Although the ADA was previously developed for SCG, recent research showed the potential of incorporating GCG in IMU-based cardiography. Our work has further validated the benefit of this combined approach to VCG analysis using both SCG and GCG. The VCG method leverages the mutually orthogonal information that can be obtained from all six degrees of freedom, thereby allowing for a comprehensive, multi-faceted analysis of cardiac vibrations. The exploitation of fundamentally different noise rejection criteria between the SCG and GCG signals through selecting results on the basis of confidence enabled a higher overall accuracy. Additionally, the utilization of feature amplification within the waveforms improved heart rate calculations as well as beat detection accuracy in comparison to a standard autocorrelation-based approach. However, note that the performance was only evaluated in scenarios involving motion artifacts from heavy chest movements caused by respiration. The reliability of the system in situations involving walking, sitting, or larger movements has not yet been determined.

Regarding heart rate measurement, the limitations of averaged HR calculations motivated an expansion of the algorithm functionality for beat detection in which the AO point was used as a timing indicator of the heartbeat. The heart rate measurements obtained from the VCG waveform within a specific window produced relatively accurate results when referenced with the equally weighted RR-intervals. However, the nature of the windowed autocorrelation analysis limited the quality of measurement to an averaged result within each timeframe. Such a smoothed measurement was not aligned with each beat and additionally beat detection was preferentially weighted toward producing FP points. In other words, performing the same measurement over multiple windows and multiple overlapping timeframes predicted an order of magnitude more beats than existed, which increased the likelihood of a FP detection. These inaccuracies were circumvented by using the gyration signal as a method to filter false AO points. The implementation of an AO detection algorithm improved heart rate measurement accuracy from a $r^{2}$ value of 0.9783 to 0.9982 and was found to be especially beneficial in cases of high HRV.

Although this detection method had better overall performance, it was limited in some cases. First, the method was dependent on the accuracy of the averaged heart rate obtained from autocorrelation. Outliers in this averaged heart rate measurement would propagate and erroneously bias the instantaneous detection algorithm. Hence despite the fact that the outlier filtration functionality was developed rigorously through an exertion study, the outliers misinformed beat detection. However, since the AO detection was developed as merely a refinement tool in improving the instantaneous heart rate, this was an anticipated limitation.

An additional limitation of VCG sensing was in its sensitivity to accurate sensor placement [58,59]. In our work, all of the sensor readings were obtained from a consistent sensor placement performed by an experienced technician. However, this did not eliminate the possibility of human error. The algorithm was optimized based on the waveform detected by the IMU at a specific point; therefore, a change in position had the potential to modify the waveform detrimentally with respect to the VarWin amplification. For example, while most datasets had a TPR of above 98%, some failed with up to a third of the beats missed. This was speculated to be due to sensor displacement causing unfamiliar waveforms. An extension of this work could include an efficacy study that characterized the waveform dependence on sensor placement.

Furthermore, a large divergence in heart rate measurements was seen in Figure 6 and Figure 7 around the median range from 40 to 90 bpm when compared with lower or elevated heart rates. Although a smaller number of inaccurate measurements were simply due to a lack of experimental data at the extremes, the fraction of these inaccuracies was also lower. This improved detection accuracy at the extremes was consistent with both averaged and instantaneous heart rate measurements for the SCG and GCG (and therefore VCG) signals. It was therefore attributed to a limitation of the algorithm architecture caused by adaptive thresholding of the AO peaks to prevent crosstalk from any AC peaks that exhibited a similar morphology. The waveform morphology of the S1 and S2 sounds was less distinct at median heart rates, which interfered with AO peak identification causing the left ventricular ejection time to be mistaken as a BTB interval. Note that while larger cardio-mechanical movement at elevated heart rates could improve peak detection accuracy, it would not affect the accuracy at lower heart rates. Additionally, the motion artifact from concurrently heavier respiratory activity would negate any increase in the signal to noise ratio caused by higher waveform amplitudes or better distinctions between the S1 and S2 sounds.

The GCG waveform offered a valuable verification tool for SCG AO detection. Strong similarities between the AO and mitral opening (MO) morphologies in the SCG waveform induced FP detection at the rapid ejection (RE) and AC points. However, waveforms in the $g_{X}$ axis did not exhibit such morphology, which enabled a discrimination of AO and MO peaks using gyration. This significantly reduced the number of incorrectly detected peaks due to the S2 region. Such fundamentally different noise rejection criteria, especially for the S2 sound as mentioned in Section 3.2 and visualized in Figure 4, were exploited through the inclusion of the $g_{X}$ axis to verify AO peak detection in the SCG signal. In addition to the statistical results, these characteristics establish the superiority of a VCG detection scheme over either SCG or GCG alone.

## 6. Conclusions

On the road to remote health care, our research contributes to filling the gap between gold standard, clinically validated health monitors that are complex or impractical, and current biometric wearables that are inaccurate or expensive. In this context, we have developed an electro-mechanical cardiac monitor that costs less than $100 and can be assembled and used at home on a stationary, supine subject. The monitor delivered measurements of averaged and instantaneous heart rate for such subjects at rest and under physical exertion. Cardiac mechanical activity was measured by an upgraded version of the ADA [33] that processed cardiac-induced sternal vibrations as VCG signals. The measurements were referenced with ECG that measured the electrical activity of the heart using the standard Pan Tompkins [57] algorithm for the ECG signal analysis. Data acquisition was validated by an external sphygomanometer. For the purpose of VCG signal processing, the ADA functionality was upgraded by first modifying the SCG analysis for GCG, and then extending the decision-making algorithms to account for both SCG and GCG. At a sampling rate of 200 Hz, averaged heart rate measurements using the ADA on SCG, GCG, and VCG resulted in squared correlation coefficients of 0.9482, 0.9305, and 0.9783, respectively when compared with ECG measurements. In an extension of the algorithm, cardiac beats were identified with a TPR of 96.6% by leveraging averaged heart rate measurements. This produced an effectively instantaneous heart rate measurement that resulted in a $r^{2}$ correlation coefficient of 0.9982 with its ECG reference. The algorithm was able to perform each round of measurements in 88 ms on a standard desktop computer. Its efficiency attested to its potential as an electro-mechanical cardiac monitoring solution for stationary, supine subjects in clinically relevant applications. The true value of the overall system is in its simplicity and affordability, whilst still being unobtrusive, non-invasive, real-time, and accurate. Even outside of clinical settings, the content-rich data obtainable from continuous monitoring and the detailed mapping of cardiac trends by using diffused cost-effective monitors such as this, would present a new horizon in health tracking and prediction.

## Figures and Tables

**Table 1:** AO detection results for number of beats, true positive beats, false positive beats, false negative beats, true positive rate, positive prediction rate, and heart rate *r*^2^ coefficient.

Type	File	# Beats	TP	FP	FN	TPR	PPV	*r* ^2^
Rest	1	478	478	0	0	1	1	0.9983
	2	505	505	0	0	1	1	0.9959
	3	565	537	1	28	0.9504	0.9981	0.9773
	4	584	577	0	7	0.988	1	0.9945
	5	569	568	4	1	0.9982	0.993	0.9949
	6	437	437	6	0	1	0.9865	0.9997
	7	449	438	0	11	0.9755	1	0.9952
	8	539	501	11	38	0.9295	0.9785	0.9924
	9	353	353	0	0	1	1	0.9978
	10	527	518	0	9	0.9829	1	0.9757
	11	466	466	0	0	1	1	0.9962
	12	450	450	0	0	1	1	0.9931
	13	577	574	0	3	0.9948	1	0.8487
	14	395	395	0	0	1	1	0.9995
	15	505	498	1	7	0.9861	0.998	0.9223
	16	438	400	12	38	0.9132	0.9709	0.9724
	17	316	316	0	0	1	1	0.9975
	18	461	457	0	4	0.9913	1	0.9982
	19	323	302	0	21	0.935	1	0.9987
	20	531	523	0	8	0.9849	1	0.9958
	21	511	511	1	0	1	0.998	0.9972
	22	508	419	0	89	0.8248	1	0.9293
	23	502	460	2	42	0.9163	0.9957	0.9996
	24	423	423	0	0	1	1	0.9983
	25	496	324	0	172	0.6532	1	0.9966
Recovery	1	656	655	1	1	0.9985	0.9985	0.9707
	2	655	651	0	4	0.9939	1	0.9991
	3	577	520	0	57	0.9012	1	0.8099
	4	699	699	1	0	1	0.9986	0.9975
	5	752	717	1	35	0.9535	0.9986	0.9837
	7	483	473	1	7	0.9854	0.9979	0.9986
	9	583	579	0	4	0.9931	1	0.9994
	10	620	607	0	13	0.979	1	0.994
	11	682	678	0	4	0.9941	1	0.9949
	12	464	463	1	1	0.9978	0.9978	0.9986
	13	655	648	0	7	0.9893	1	0.9964
	14	578	577	0	1	0.9983	1	0.9988
	16	502	420	5	82	0.8367	0.9882	0.9979
	17	382	381	0	1	0.9974	1	0.9996
	18	482	468	11	14	0.971	0.977	0.9986
	19	430	421	0	9	0.9791	1	0.8693
	20	664	601	4	63	0.9051	0.9934	0.9603
	21	634	621	11	13	0.9795	0.9826	0.986
	22	364	364	1	0	1	0.9973	0.9955
	24	471	455	0	16	0.966	1	0.9994
	25	743	734	0	9	0.9879	1	0.9942
Total	46	23,984	23,162	75	819	0.9657	0.9968	0.9982
